# Phylogenomic insights from the complete chloroplast genome of *Berchemiella wilsonii* var. *pubipetiolata* H. Qian (Rhamnaceae) from Zhejiang

**DOI:** 10.1080/23802359.2026.2635835

**Published:** 2026-02-27

**Authors:** Yingchao Dai, Dongbin Li, Hong Zhu

**Affiliations:** ^a^ Administration of Zhejiang, Qingliangfeng National Nature Reserve, Hangzhou, China; ^b^ Ningbo Forestry Development Center, Ningbo, China; ^c^ Zhejiang Academy of Forestry, Hangzhou, China

**Keywords:** *Berchemiella wilsonii* var. *pubipetiolata*, chloroplast genome, phylogenetic analysis

## Abstract

*Berchemiella wilsonii* var. *pubipetiolata* H. Qian, a member of the Rhamnaceae family, is endemic to mountainous regions in two eastern Chinese provinces. Here, we present the first assembly of its chloroplast (cp) genome, which exhibits a typical quadripartite structure, spans 160,317 bp long and has a 37.20% GC content. Genome annotation identified 131 genes, including 84 messenger RNA (mRNA) genes, 37 transfer RNA (tRNA) genes, and eight ribosomal RNA genes. Phylogenetic analysis confirms its close relation to *B. wilsonii,* thereby providing valuable genomic resources for conservation efforts and evolutionary studies of East Asian *Berchemiella* species.

## Introduction

*Berchemiella wilsonii var. pubipetiolata* H. Qian 1988, as a variant of *Berchemiella wilsonii* (C. K. Schneid.) Nakai 1923. This species is only found in two disjunct regions: the north-eastern Dabie Mountain in Anhui Province and the western Tianmu Mountain in Zhejiang Province. Given its restricted distribution, *B. wilsonii* var. *pubipetiolata* has been designated as a Zhejiang Provincial Key Protected Species. Beyond its status as a protected species, *B. wilsonii* var. *pubipetiolata* holds significant economic and ornamental value due to its high-density timber, decorative red fruiting inflorescences, and unique bark patterns. Scientifically, its distinct drupe and seed morphology are critical for understanding taxonomic boundaries and evolutionary transitions within Rhamnaceae (Wei et al. 2014).

Current research primarily focuses on its population structure, dynamic characteristics (Hu et al. 2005; Pang et al. 2025) and genetic diversity. Specifically, studies using AFLP markers have explored its genetic variation (Kang et al. 2006; Kang et al. 2008). Chloroplast (cp) genomes have been widely recognized as powerful tools for phylogenetic inference, DNA barcoding, genome evolution study, and species conservation strategies (Wen et al. 2021; Zhu and Li 2024). However, little research has been conducted on its genome, particularly those involving the cp genome. To clarify the taxonomic and evolutionary relationships within the genus *Berchemiella*, we sequenced and assembled the complete cp genome of *B. wilsonii* var. *pubipetiolata*, and conducted comparative genomics analyses to characterize its genomic features and phylogenetic position. These findings provide valuable insights for future studies on phylogenetic and genetic diversity within *Berchemiella* species.

## Materials and methods

### Plant materials

**Figure 1.**
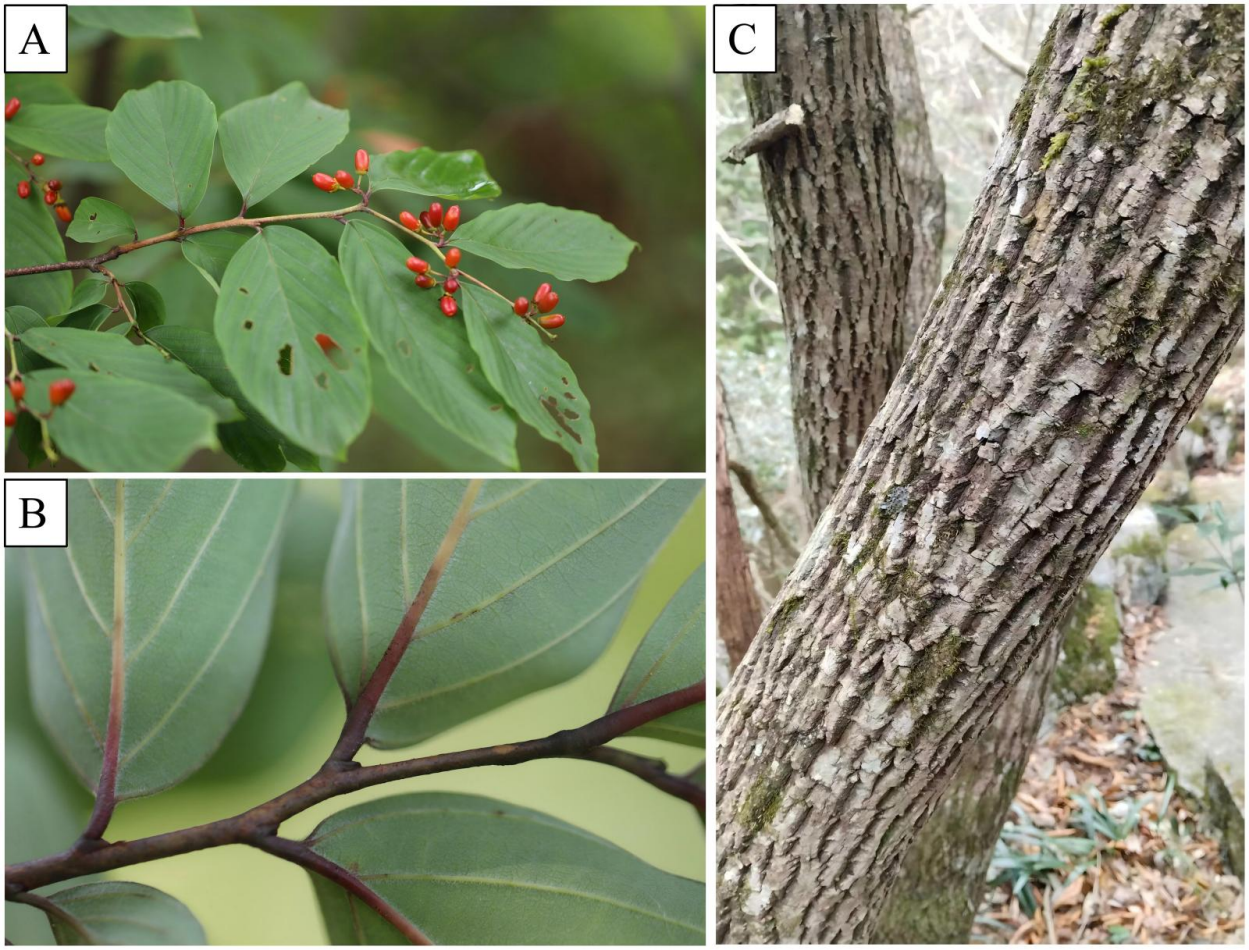
Photographs of *Berchemiella wilsonii var. pubipetiolata* showing typical morphological characteristics. (A) Red fruiting inflorescence; (B) abaxial leaf surface; (C) gray vertically cracked bark. The photo was taken by the corresponding author, Hong Zhu. The species is characterized by the abaxial surface of leaves being densely covered with short pubescence and pubescent petioles, which distinguishes it from the original variety.

Fresh leaves of *B. wilsonii* var. *pubipetiolata* were collected from Shilin, Zhejiang Qingliangfeng National Nature Reserve, Hangzhou, Zhejiang Province, China (coordinates: 30.1096°N, 118.9012°E; altitude: 904 m) in July 2025 (Figure 1). Varietal authentication was performed based on validated morphological descriptors from original diagnosis by Qian (1988) and the Flora of China (Chen and Schirarend 2007). Specifically, this variety is distinguished from the original variety (*B. wilsonii* var. *wilsonii*) by its petioles and the abaxial surface of the leaf blades being densely covered with short pubescence, whereas the original variety is typically glabrous or nearly so. The specimen was deposited in the Herbarium of Zhejiang Academy of Forestry (HZJAF, contact: Hong Zhu, Email: zhuhong@zjforestry.ac.cn) under the voucher number HZ2025-QLF-03.

### DNA extraction and sequencing, genome assembly, and annotation

Total genomic DNA was extracted using a plant DNA kit (Tiangen Biotech, Beijing, China). The quality and integrity of the extracted DNA were initially evaluated via agarose gel electrophoresis. Subsequently, the DNA was sheared into fragments using ultrasonication. Libraries were constructed and subjected to quality control, after which qualified samples were sequenced using the Illumina NovaSeq 6000 platform (San Diego, CA) with paired-end sequencing (PE150). Raw reads were processed for quality filtering using fastp v. 0.23.4 (Chen 2023) and followed by genome assembly with SPAdes v. 3.10.1 (Bankevich et al. 2012). Organelle genome annotation was conducted using GetOrganelle v.1.7.7.1 (Jin et al. 2020). The circular cp genome and cis/trans-splicing gene maps were generated using CPGView (Liu et al. 2023).

### Phylogenetic analysis

For phylogenetic reconstruction, the cp genome sequences of 17 related taxa were retrieved from the GenBank database. These taxa belong to the family Rhamnaceae and include three species from the genus *Berchemiella* Nakai, three from *Berchemia* Neck. ex DC., one from *Sageretia* Brongn., three from *Rhamnus* L., one from *Paliurus* Mill., three from *Ziziphus* Mill., and three from *Hovenia* Thunb. Additionally, *Hippophae rhamnoides*, *H. tibetana*, and *Elaeagnus macrophylla* were selected as outgroup taxa for phylogenetic analysis. Complete plastome sequences were utilized using the program MAFFT v. 7.429 (Katoh et al. 2019) with circular sequences adjusted to the same starting position. Phylogenetic analysis was performed using software RAxML v. 8.2.12 (Stamatakis 2014) to construct a maximum-likelihood (ML) tree. The analysis was conducted under the GTR substitution model with 1000 bootstrap replicates to evaluate node support.

## Results

**Figure 2.**
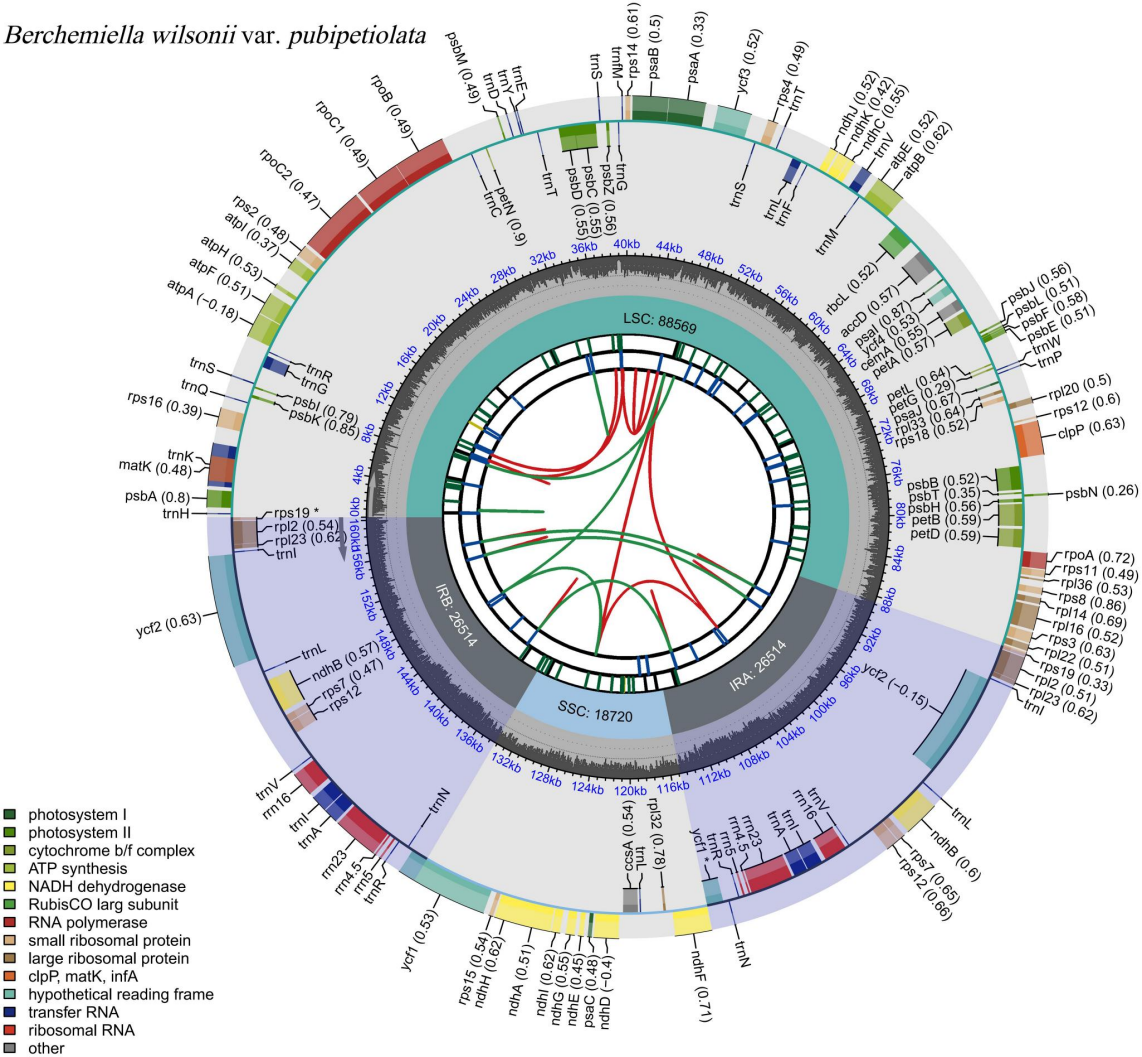
The CPGview-generated circular map of the cp genome of *Berchemiella wilsonii* var. *pubipetiolata* features six default annotation tracks. The first track, radiating outward from the center, displays dispersed repeats, including direct (D) and palindromic (P) repeats, represented by red and green arcs, respectively. The second track depicts long tandem repeats as short blue bars, while the third track shows short tandem repeats or microsatellites using color-coded bars of varying lengths. The fourth track delineates the genome’s structural organization, comprising the small single-copy (SSC), inverted repeat (IRa and IRb), and large single-copy (LSC) regions. The fifth track illustrates the GC content across the genome, and the sixth track presents genes color-coded according to their functional categories. Codon usage bias, where present, is indicated in parentheses following gene names. Genes transcribed in the clockwise direction are positioned on the inner side of the track, whereas those transcribed counterclockwise are located on the outer side. A legend detailing the functional classification of genes is provided in the lower left corner.

After quality control, the obtained Clean Data contained 21,928,011 reads and 6,578,403,300 bases, with Q20 and Q30 being 98.72% and 95.39%, respectively. The total length of this cp genome is 160,317 bp, with an average sequencing depth of 1418.15× and GC content of 37.20% (Figure 2, Figure S1). A large single-copy (LSC: 88,569 bp), a small single-copy (SSC: 18,720 bp), and a pair of identical inverted repeats (IRs: 26,514 bp) regions made up the typical quadripartite structure of the genome. The base compositions of the cp genome were uneven (31.04% A, 18.93% C, 18.27% G, and 31.76% T). A total of 131 unique genes were annotated, including 37 transfer RNA (tRNA) genes, eight ribosomal RNA (rRNA) genes, 84 messenger RNA (mRNA) genes, and two pseudogenes (*rps*19 and *ycf*1). Specifically, 15 genes harbored a single intron (including *ndh*A, *ndh*B, *pet*B, *pet*D, *atp*F, *rpl*16, *rpl*2, *rps*16*, rpo*C1, *trn*A-UGC, *trn*G-UCG, *trn*I-GAU, *trn*K-UUU, *trn*L-UAA, and *trn*V-UAC), while three genes (*rps*12, *clp*P, and *ycf*3) contained two introns. Thirteen cis-splicing genes and one trans-splicing gene, *rps*12 with three unique exons, were identified (Figures S2 and S3). Additionally, the cp genome sequence of *B. wilsonii var. pubipetiolata* has been deposited in the NCBI database under the accession number PX776296.

**Figure 3.**
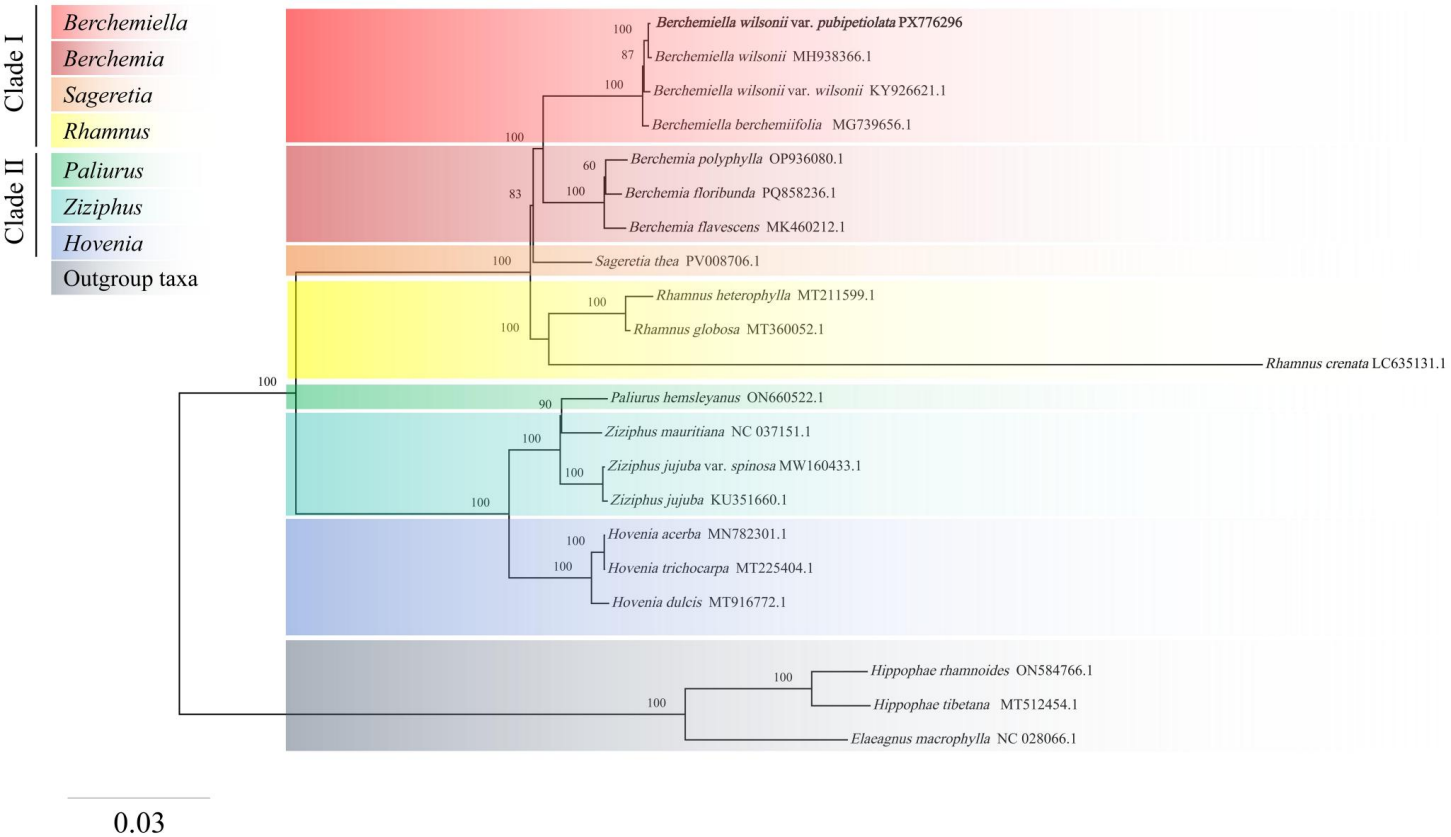
Maximum-likelihood (ML) phylogenetic tree of *B. wilsonii* var. *pubipetiolata* (shown in bold font; GenBank accession number PX776296) with 20 other taxa (designating *Hippophae rhamnoides*, *H. tibetana*, and *Elaeagnus macrophylla* as outgroup taxa) was constructed based on the complete cp genome sequence. Nodal values represent ML bootstrap (BS) values. The phylogram without species names was placed in the top left corner, with the distance bar implied by the ML method. The sources of plastid genome data are as follows: *B. wilsonii* MH938366.1 (Li et al. 2019), *B. wilsonii* var. *wilsonii* KY926621.1 (Wang et al. 2018), *B. berchemiifolia* MG739656.1 (Cheon et al. 2018), *B. polyphylla* OP936080.1, *B. floribunda* PQ858236.1, *B. flavescens* MK460212.1 (Zhu et al. 2019), *Sageretia thea* PV008706.1, *Rhamnus heterophylla* MT211599.1 (Li, Chen, Chen 2020), *R. globosa* MT360052.1 (Xie et al. 2020), *R. crenata* LC635131.1 (Wang and Yang 2021), *Paliurus hemsleyanus* ON660522.1, *Ziziphus mauritiana* NC_037151.1, *Z. jujuba* var. *spinosa* MW160433.1, *Ziziphus jujuba* KU351660.1 (Ma et al. 2017), *Hovenia acerba* MN782301.1 (Yin et al. 2020), *H. trichocarpa* MT225404.1 (Li, Ye, Bi 2020), *H. dulcis* MT916772.1 (Liu et al. 2021), *Hippophae rhamnoides* ON584766.1, and *H. tibetana* MT512454.1, *Elaeagnus macrophylla* NC_028066.1 (Choi et al. 2015).

Based on cp genomes, a phylogenetic tree was reconstructed for 18 species within the family Rhamnaceae (Figure 3). The topological structure of the phylogenetic tree showed that all species could be divided into two major clades. The first clade (clade I) included species from the genera *Berchemiella*, *Berchemia*, *Sageretia*, and *Rhamnus,* while the second clade (clade II) contained species from *Paliurus*, *Ziziphus*, and *Hovenia. B. wilsonii* var. *pubipetiolata* was positioned at the tip of *Berchemiella* clade, sharing the closest phylogenetic relationship with its congener. It also indicated that *B. wilsonii* var. *pubipetiolata* clustered together with *B. wilsonii* with high support (BS value = 100). Besides, the *Berchemiella* clade is monophyletic and sister to the *Berchemia* clade.

## Discussion and conclusions

*Berchemiella* is a genus endemic to East Asia; however, the phylogenetic relationships of *Berchemiella* and its closely related genera, such as *Berchemia*, remained controversial (Wei et al. 2014). Furthermore, the taxonomic boundaries within *Berchemiella* are still unclear due to lack of phylogenomic evidence (Wang et al. 2018; Li et al. 2019). For instance, the variety *B. wilsonii* var. *pubipetiolata* has been described solely based on a single morphological character (Qian 1988). Consequently, insufficient genomic sequence data have hindered a comprehensive understanding of the phylogeny and species identification within *Berchemiella*. In this study, the complete cp genome of *B. wilsonii* var. *pubipetiolata* was successfully assembled and annotated for the first time. The results confirm its close phylogenetic affinity with *B. wilsonii* and reveal the evolutionary relationship between the genera *Berchemiella* and *Berchemia*. This study provides new cp genome resources for *B. wilsonii* var. *pubipetiolata* and will be valuable for taxonomic revision, conservation strategies, and population genetic studies of Rhamnaceae in the future.

## Supplementary Material

Clean copy of manuscript.doc

Supplementary Materials.doc

## Data Availability

The genome sequence data that support the findings of this study are openly available in GenBank (NCBI, https://www.ncbi.nlm.nih.gov) under the accession no. PX776296. The associated BioProject, SRA, and BioSample accession numbers are PRJNA1370622, SRR36613934, and SAMN53474106, respectively.
