# Supplementary material for: Phylogenomic insights from the complete chloroplast genome of Berchemiella wilsonii var. pubipetiolata H. Qian (Rhamnaceae) from Zhejiang
Source: Mitochondrial DNA B Resour. 2026 Feb 27;11(4):457–61. doi: 10.1080/23802359.2026.2635835 (PMC12951654; doi:10.1080/23802359.2026.2635835)
Supplement: Supplementary Materials.doc [file TMDN_A_2635835_SM8812.doc]

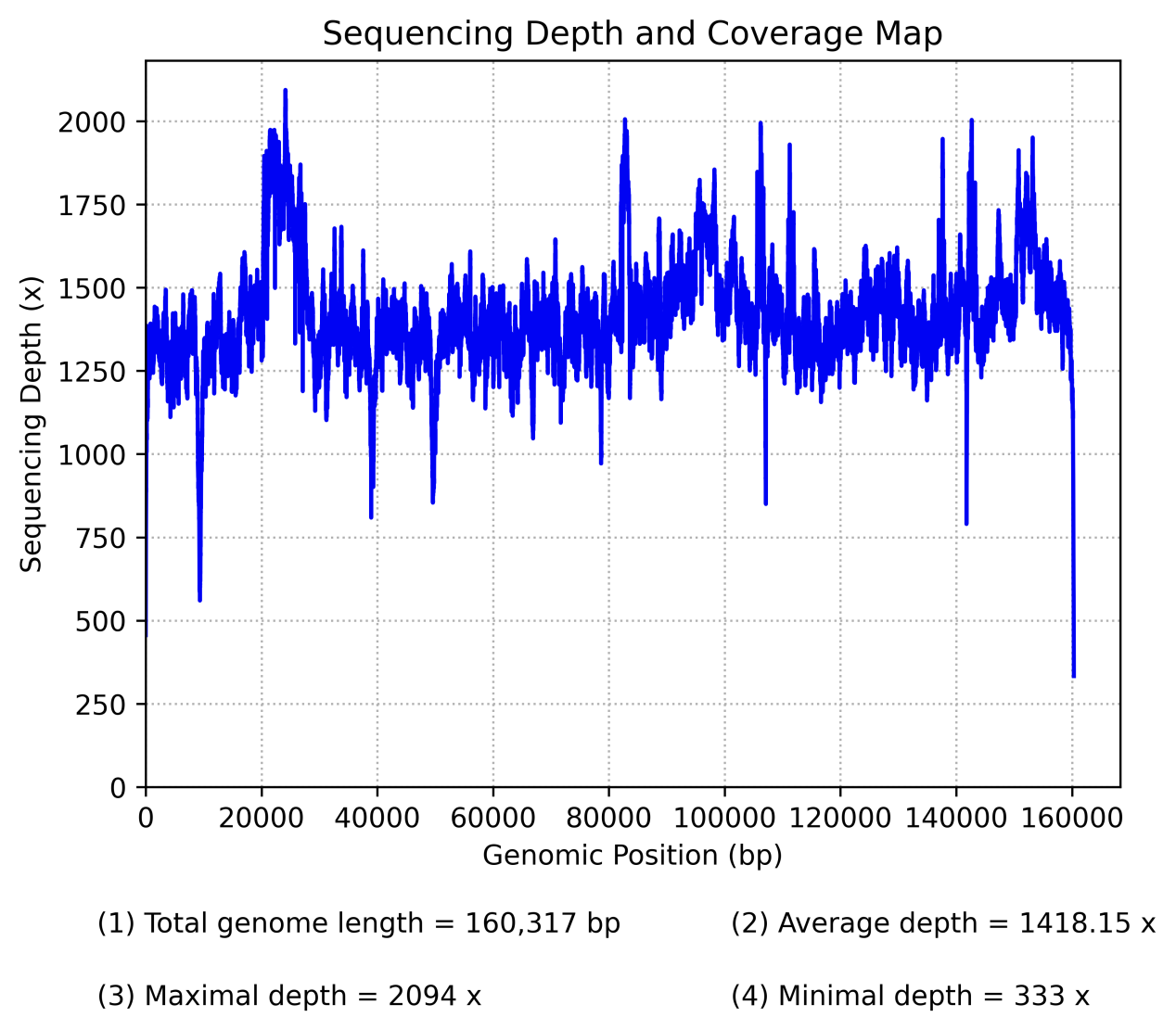


**Figure S1.** The map of sequencing depth and coverage, representing the sequencing depth on the complete chloroplast genome. The maximum sequencing depth is 2094×, the, the minimal sequencing depth is 333×, and the average sequencing depth is 1418.15×.


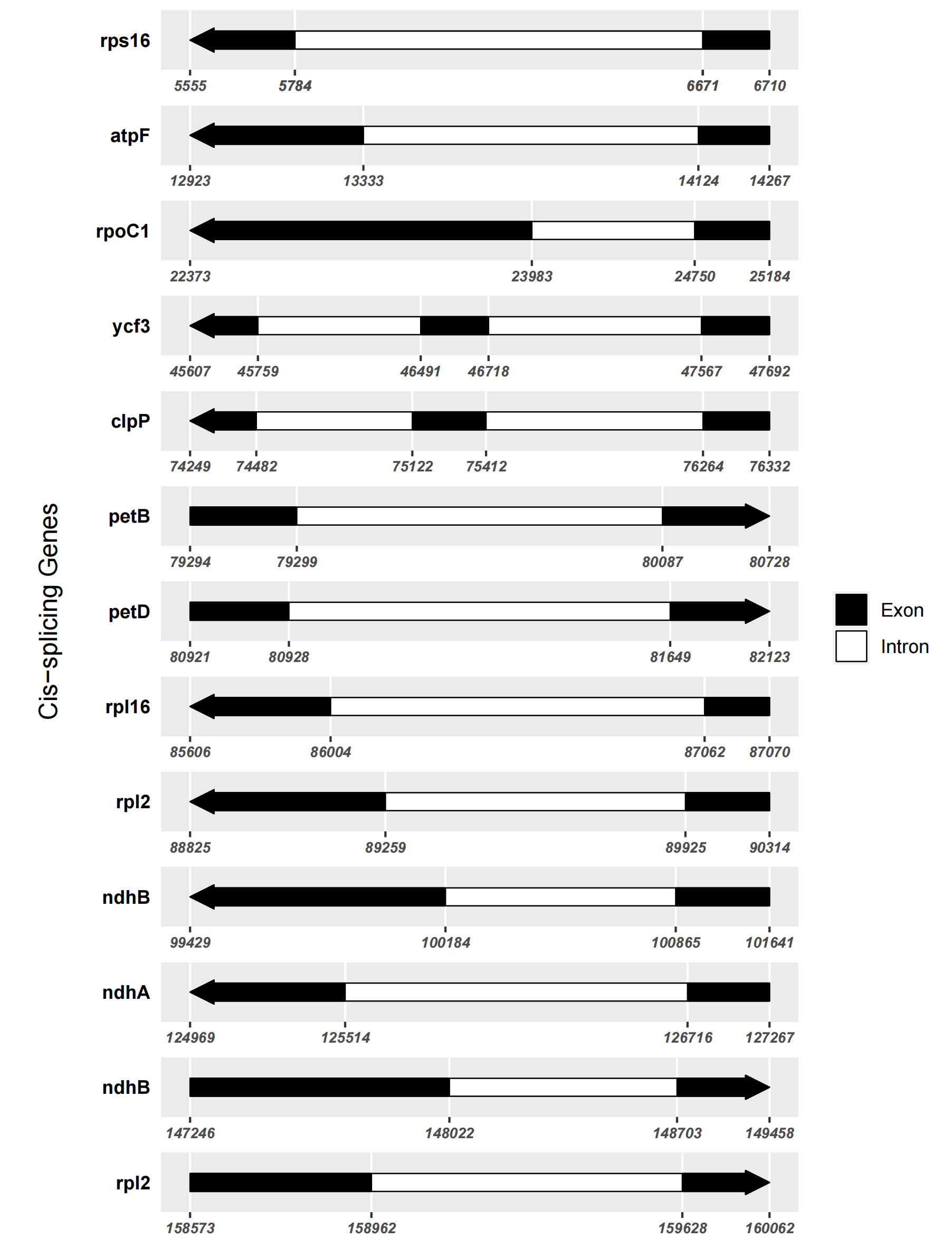
**Figure S2.** Schematic map of the cis-splicing genes in the chloroplast genome. The genes are arranged from top to bottom based on their order on the chloroplast genome. The gene names are shown on the left, and the gene structures are on the right. The exons are shown in black; the introns are shown in white. The arrow indicates the sense direction of the gene.


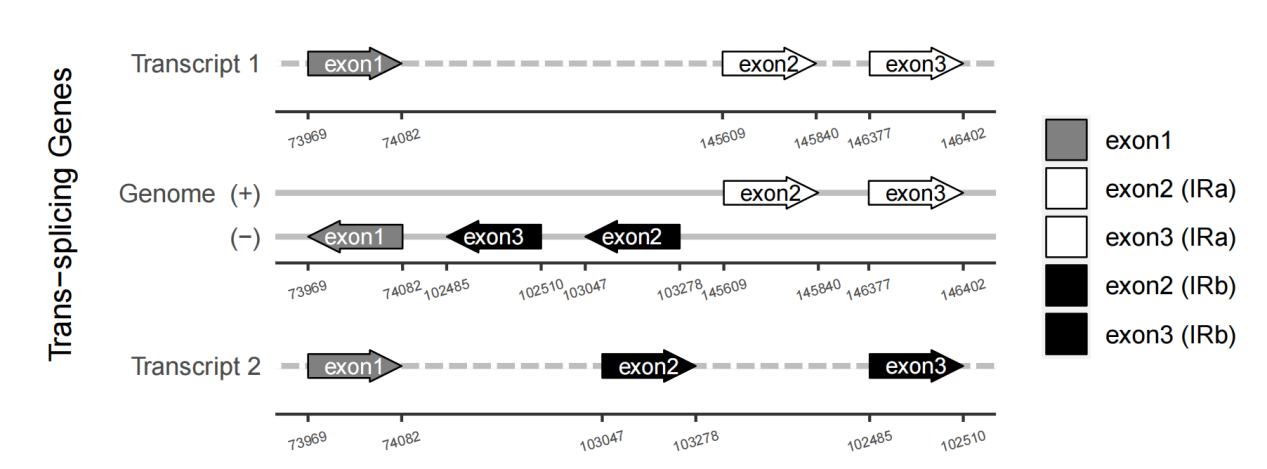
**Figure S3.** Schematic map of the trans-splicing gene *rps12* in the chloroplast genome. It has three unique exons, and two of them are duplicated as they are located in the IR regions.
